# Variations in Provision of Long-Acting Reversible Contraception Across Veterans Health Administration Facilities

**DOI:** 10.1007/s11606-023-08123-5

**Published:** 2023-06-20

**Authors:** Siobhan Mahorter, Kavita Vinekar, Jonathan G. Shaw, Maria K. Mor, Zoe H. Pleasure, Lori M. Gawron, Lisa S. Callegari

**Affiliations:** 1 Health Services Research & Development (HSR&D) Seattle-Denver Center of Innovation for Veteran Centered & Value Driven Care, Department of Veterans Affairs (VA) Puget Sound Health Care System, Seattle, WA USA; 2 VA HSR&D Center for the Study of Healthcare Innovation, Implementation & Policy, VA Greater Los Angeles Healthcare System, Los Angeles, CA USA; 3 Department of Obstetrics & Gynecology, David Geffen School of Medicine, University of California, Los Angeles, Los Angeles, CA USA; 4 VA HSR&D Center for Innovation to Implementation, VA Palo Alto Health Care System, Palo Alto, CA USA; 5 Division of Primary Care & Population Health, Stanford University School of Medicine, Stanford, CA USA; 6 VA HSR&D Center for Health Equity Research and Promotion, VA Pittsburgh Healthcare System, Pittsburgh, PA USA; 7 Department of Biostatistics, University of Pittsburgh Graduate School of Public Health, Pittsburgh, PA USA; 8 Department of Health Systems & Population Health, University of Washington School of Public Health, Seattle, WA USA; 9 Health Services Research & Development, VA Salt Lake City Health Care System, Salt Lake City, UT USA; 10 Department of Obstetrics & Gynecology, University of Utah, Salt Lake City, UT USA; 11 Department of Obstetrics & Gynecology, University of Washington School of Medicine, Seattle, WA USA

## INTRODUCTION

Equitable access to the full range of contraceptive methods – including long-acting reversible contraception (LARC) – is a critical component of high-quality reproductive healthcare.^1^ This is particularly true for the growing number of reproductive-age Veterans Health Administration (VA) users, who may face elevated risks from unwanted or mistimed pregnancy due to a high burden of medical and mental health comorbidities.^2,3^ Placement of LARC methods, including intrauterine devices (IUDs) and contraceptive implants, requires specialized training routinely held by gynecologists but more variably by primary care clinicians. Within VA, LARC provision is of particular concern in facilities that have limited gynecology care, such as community-based outpatient clinics (CBOCs) which are primarily staffed by primary care clinicians, and rural VA Medical Centers (VAMCs).^4^ Although VAMC or CBOC facilities may also contract with community care clinicians for LARC provision, many rural areas lack providers in the community who are skilled in LARC placement.^4,5^ As limited data have been published on LARC provision within VA to date, we used VA administrative data to examine variations in rates of LARC provision and clinician type placing LARC, comparing facilities by rurality and facility type.

## METHODS

We obtained administrative data for all female Veterans of reproductive age (18–44) seen at VA for primary or gynecology care in 2019. We constructed an “at risk of pregnancy” cohort by excluding those with evidence of prior sterilization, hysterectomy, diagnosed infertility, or birth or pregnancy at the end of 2019.^6^ LARC provision was captured with billing codes, location and clinician type with VA station and clinician codes, and facility-level rurality with rural-urban commuting area codes. We calculated the proportion of facilities with “any LARC provision,” defined as evidence of  ≥3 IUDs or  ≥3 implants placed on-site at the facility in 2019. We examined variations across facilities in proportion of Veterans at risk of pregnancy who were provided a LARC in 2019 and proportion of LARCs placed by non-gynecologists [median, interquartile range (IQR)].

## RESULTS

**Figure 1:**
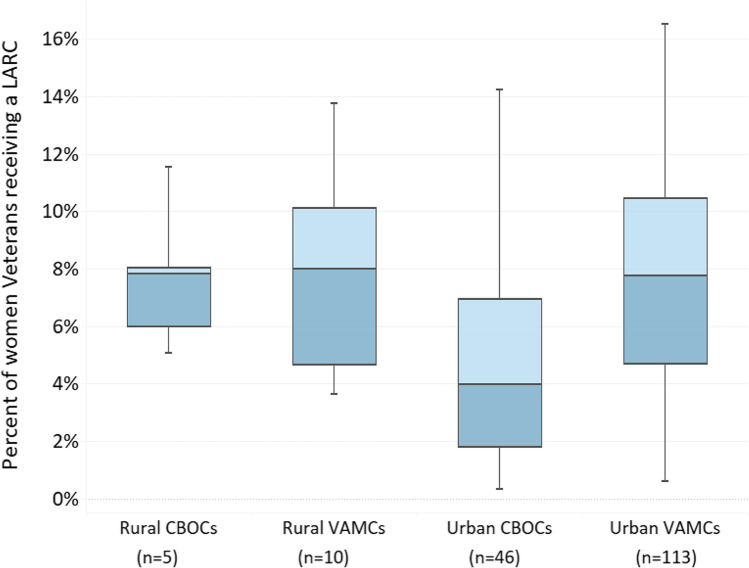
Variation in percent of women Veterans provided a long-acting reversible contraceptive (LARC) method among VA facilities placing LARC in 2019, by rurality and facility type. CBOC: Community-based outpatient clinic VAMC: VA Medical Center.

Among our cohort of 167,558 Veterans, 6,824 (4.1%) had an IUD and 2,877 (1.7%) had an implant placed in 2019, with only 4.2% of LARC methods placed through community care. Urban VAMCs were most likely to provide any LARC (113/145;78%), followed by rural VAMCs (10/25;40%), urban CBOCs (46/432;11%) and rural CBOCs (5/269;2%). Among rural VAMCs and CBOCs that placed LARC, median rates of LARC provision [8.0% (IQR = 4.7‒10.1) and 7.8% (IQR = 6.0‒8.0), respectively] were comparable to rates observed in urban VAMCs (median = 7.8%;IQR = 4.7–10.4; see Fig. 1). Rates of LARC provision were lower among urban CBOCs (median = 4.0%;IQR = 1.8‒6.9). Overall, a third of LARC methods were placed by non-gynecologists (33%). Median proportions of LARC placed by non-gynecologists varied by facility type and location, with 100% at rural CBOCs, 51% (IQR = 2‒100%) at urban CBOCs, 38% (IQR = 5‒100%) at rural VAMCs, and 8% (IQR = 2‒42%) at urban VAMCs.

## DISCUSSION

We found substantial variation in LARC provision across facilities, with low provision in many facilities raising concerns about gaps in contraceptive access. Consistent with prior studies using VA organizational survey data,^4^ provision of any LARC was substantially less frequent in rural facilities. However, among the minority of rural facilities placing LARC, rates of provision were comparable to those in urban VAMCs, demonstrating that on-site access to LARC is achievable in rural clinics. Lower levels of LARC provision among urban CBOCs may reflect closer proximity to VAMCs and community clinicians offering the service. The finding that over 20% and 80%, respectively, of urban VAMCs and CBOCs did not provide any LARC suggests gaps even in urban areas where staffing and patient volumes are generally more favorable. The sizeable proportion of LARCs placed within VA by primary care clinicians suggests that ongoing investment in LARC training for non-gynecologists or hiring of primary care clinicians with existing expertise in gynecologic procedures could be promising strategies for expanding access. Additional work is needed to explore modifiable determinants of LARC provision across facility type and geography to inform strategies for addressing gaps. One important limitation of this work is the absence of Veterans’ perspectives and needs, which will be essential to ensure efforts to expand contraceptive access are patient-centered and equitable. Given growing restrictions on reproductive healthcare across much of the US, VA’s attention to ensuring equitable contraceptive access will be critical to safeguarding Veterans’ autonomy and health.
